# Thermodynamics of Tl_2_PrBr_5_ Compound and Re-examination of Phase Equilibria in the PrBr_3_–TlBr System

**DOI:** 10.1021/acsomega.4c07419

**Published:** 2024-12-06

**Authors:** Beata Salamon-Baran, Jan Kapała, Leszek Rycerz, Irena Szczygieł

**Affiliations:** † Department of Inorganic Chemistry, Faculty of Production Engineering, Wroclaw University of Economics and Business, Komandorska 118/120, 53-345 Wrocław, Poland; ‡ Division of Analytical Chemistry and Analytical Metallurgy, Faculty of Chemistry, Wroclaw University of Science and Technology, Wybrzeże Wyspiańskiego 27, 50-370 Wrocław, Poland

## Abstract

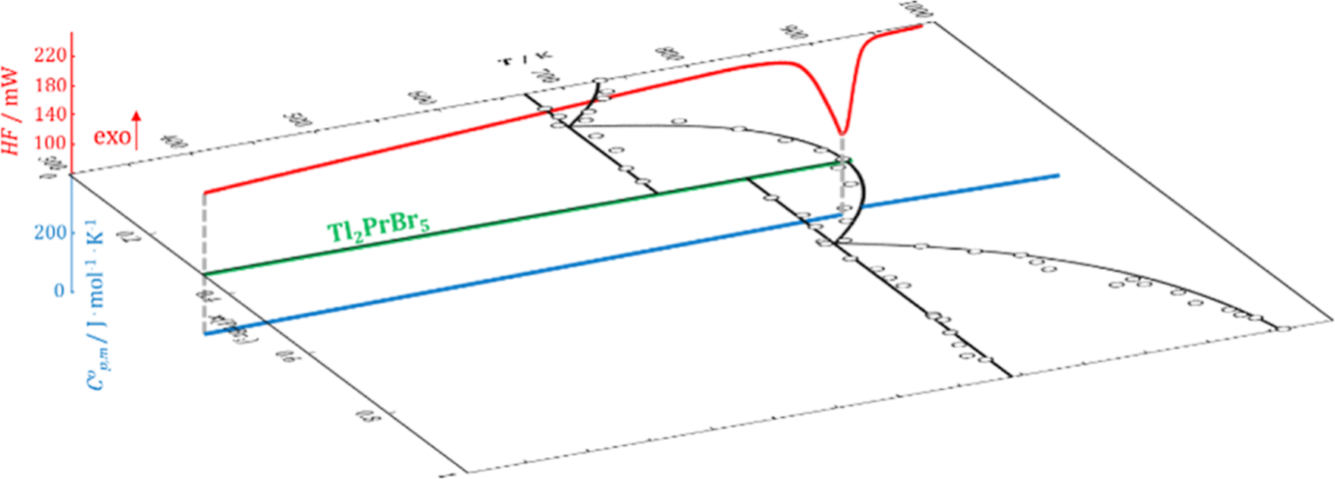

The PrBr_3_–TlBr phase diagram was first established in the 1970s. Due to some inaccuracies, it was redetermined using differential scanning calorimetry. The results obtained differ significantly from those in the literature, which has been discussed in this paper. Only one congruently melting Tl_2_PrBr_5_ was confirmed, and the thermodynamic characterization of molar heat capacity temperature dependence was carried out for this compound. The compatibility of the obtained data was examined by using the CALPHAD method. The dependences of mixing enthalpy and mixing entropy of the liquid phase on the mole fraction were estimated.

## Introduction

Essential to the work of materials engineers and metallurgists, phase diagrams enable the development of materials with specific properties and are also indispensable for controlling the heat treatment procedures of these materials. Since experimentally determining phase diagrams is relatively expensive, time-consuming, and challenging, various methods have been developed to predict the topology and properties of phase diagrams. This is particularly important for high-tech materials, which are often multicomponent systems. One such technique is CALPHAD,^1^ which is used in this work.

Due to their attractive applications in high-tech, interest in lanthanide halides and their systems with alkali metal halides has not waned over recent years.^2−5^ As technology develops, modifying existing materials to search for new ones with even more interesting properties seems natural. Meanwhile, our research, initiated several years ago on phase diagrams of lanthanide halide systems with thallium halides,^6,7^ has garnered attention in the scientific community and has been expanded to include specialized studies on applying the compounds we have identified. Thallium halide–lanthanide compounds exhibit excellent scintillation properties.^8−15^ However, an in-depth understanding of the thermodynamics of the systems from which these compounds are derived is required to continue scintillation research properly. Phase diagrams of thallium halides with lanthanide halides will not only be helpful in the identification of phases, including new ones not yet explored, but are also necessary to assess the thermal stability of the phases and select synthesis methods for the compounds.

The present work continues studying a series of LnX_3_–TlX (Ln = lanthanide, X = halide) systems.^6,7^ The PrBr_3_–TlBr phase diagram was first measured using DTA by Molodkin et al. in 1978.^16^ However, compared with this type of system, the unusual topology of the phase equilibria in the system indicated the need to verify its phase diagram. The data obtained experimentally in this work were further used to optimize this diagram using the CALPHAD method, thereby supplementing the thermodynamic description of the PrBr_3_–TlBr system with previously unmeasured dependencies of enthalpy and entropy of mixing on composition.

## Materials and Methods

The purity of compounds in thermal analysis is crucial for the quality of the obtained phase diagrams. Due to experiences related to the questionable purity of commercially available anhydrous lanthanide bromides, we opted for our synthesis of praseodymium(III) bromide, conducted using the so-called wet method. All details regarding the conditions and individual steps of the synthesis have been described in previous papers.^6,7^

As a result of the conducted synthesis, anhydrous PrBr_3_ of satisfactory purity was obtained. The amount of bromide ions controlled by means of the Mohr method was 62.95% and was close to the theoretical value (62.98%), while the praseodymium ion content was determined by complexometric titration. The obtained value of 37.01% was also close to the theoretical content (37.02%). The origin and purity of all reagents used not only for the synthesis of PrBr_3_ but also for the preparation of samples have been listed in Table 1.

**Table 1:** Source and Purity of Reagents Used in This Study

compound	CAS number	source	mass fraction purity
Pr_6_O_11_	12037-29-5	Sigma-Aldrich	0.999
HBr	10035-10-6	Sigma-Aldrich	0.99
NH_4_Br	12124-97-9	Sigma-Aldrich	0.99
PrBr_3_	13536-79-3	own synthesis	0.999
TlBr	7789-40-4	abcr GmbH	0.99999

Due to the strong hygroscopicity of lanthanide halides, all sample preparation was carried out in a glovebox under an argon-protective atmosphere. The method of obtaining and storing samples for measurements has also been described previously.^7^

**Figure 1:**
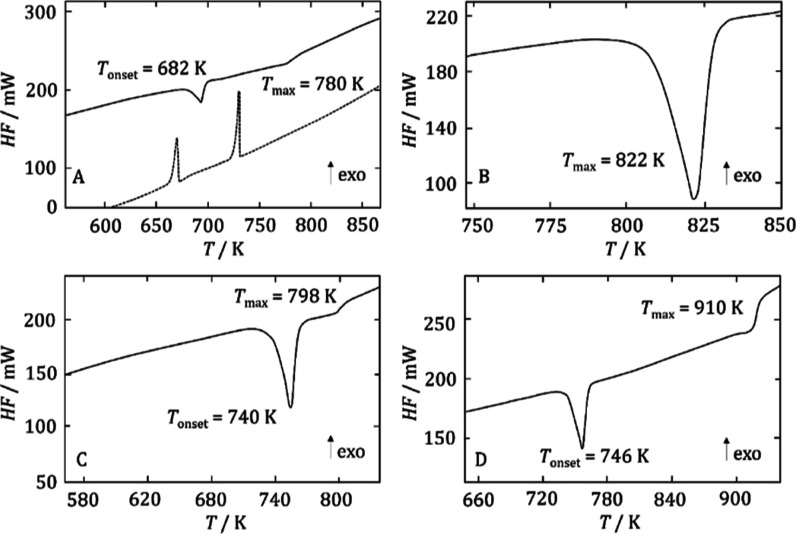
DSC heating curves (solid lines) from the PrBr_3_–TlBr system for samples with the following compositions: (A) *x*(PrBr_3_) = 0.197, (B) *x*(PrBr_3_) = 0.331, (C) *x*(PrBr_3_) = 0.602, and (D) *x*(PrBr_3_) = 0.811, recorded at a heating/cooling rate of 5 K·min^–1^. The dotted line represents the cooling curve.

The phase diagram was measured using the differential scanning calorimetry (DSC) technique with a SETARAM Labsys Evo differential scanning calorimeter. The mentioned hygroscopic nature of the compounds under study also necessitated using special quartz ampules for measurements, the diameter of which was adjusted to the measurement cells to ensure the best possible thermal contact. All heating and cooling curves were recorded at a rate of 5 K·min^–1^. Due to the overcooling effect observed in all thermograms, only heating curves were considered for constructing the diagram (dotted line in Figure 1). Temperatures of eutectic and peritectic transitions were determined as the onset temperature of the effect, while the liquidus temperature was defined as the maximum temperature of the effect. The dependence of molar heat capacity on temperature for the congruently melting compound was determined using a step method with the same apparatus, and α-Al_2_O_3_ (NIST, standard material 720) with a known molar heat capacity–temperature dependence^17^ (340–1870 K) was used as a standard
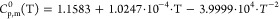
1

A detailed description of the method and conditions for measuring the dependence of molar heat capacity on temperature was included in the previous paper.^7^

## Results

**Figure 2:**
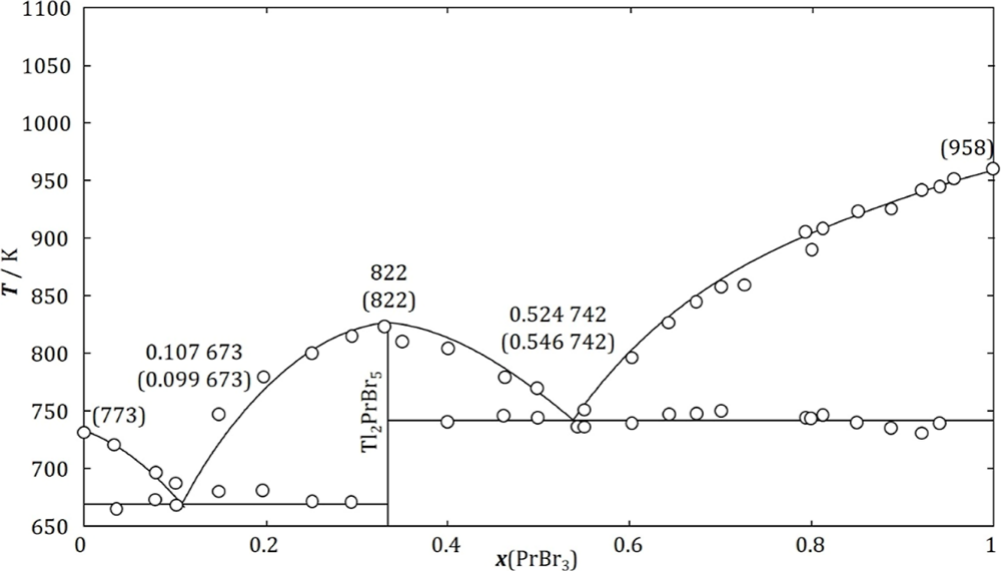
PrBr_3_–TlBr phase diagram (points—experimental data and lines and values in parentheses—data optimized using the CALPHAD technique).

Due to the controversies regarding the PrBr_3_–TlBr system developed by Molodkin et al.^16^ resulting from the number of intermediate compounds present in the system and their phase transformations occurring in a relatively narrow temperature range, the phase equilibria in this system were re-examined. The result of the measurements was the acquisition of a new phase diagram (Figure 2) based on 27 heating and cooling curves across the entire compositional range *x*(PrBr_3_) = 0–1.

The compositions of all measured samples and results of DSC measurements are given in Table 2. The measured melting temperatures of praseodymium(III) bromide and thallium bromide were 958 and 733 K, respectively, and these values are close to those known from the literature (966 K^18^ and 733 K^17^).

**Table 2:** Results of the DSC Experiments Performed on the PrBr_3_–TlBr System^a^

*x*(PrBr_3_)	*T*/K	*T*/K	*T*/K
	TlBr–Tl_2_PrBr_5_ eutectic	Tl_2_PrBr_5_–PrBr_3_ eutectic	liquidus
0.000			733
0.034	663		718
0.078	674		698
0.100	668		689
0.148	681		749
0.197	682		780
0.250	671		802
0.295	671		815
0.331			822
0.350			812
0.399		741	807
0.462		746	779
0.498		746	770
0.550		737	751
0.602		740	798
0.642		750	828
0.673		748	844
0.701		750	858
0.792		746	906
0.799		744	891
0.811		746	910
0.850		739	925
0.887		736	926
0.920		732	942
0.941		741	946
0.956			951
1.000			958

a The standard uncertainties of measured temperature and mole fraction equal 3 K and 0.5%, respectively.

In all thermograms obtained, the thermal effect occurring at the highest temperature was treated as liquidus. Two endothermic effects can be observed in the DSC curves for samples in the composition range 0 > *x*(PrBr_3_) > 0.333 (Figure 1A). The effects occurring at approximately 673 K are related to the eutectic transformation
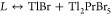
2

**Figure 3:**
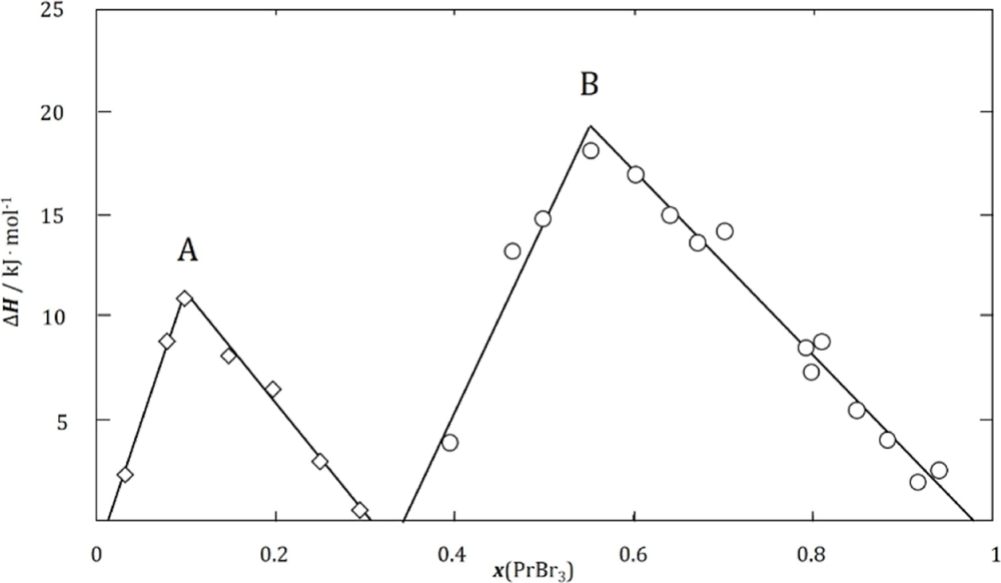
Tammann plots of the PrBr_3_–TlBr system: (A) determination of the TlBr–Tl_2_PrBr_5_ eutectic composition and (B) Tl_2_PrBr_5_–PrBr_3_ eutectic composition.

This effect is no longer visible in the DSC curves for samples corresponding to molar fractions *x*(PrBr_3_) > 0.333 (Figure 1B). The composition of the eutectic and the enthalpy associated with this transformation were determined directly from the Tammann diagram and are *x*(PrBr_3_) = 0.099 ± 0.007 and 11.0 ± 0.6 kJ·mol^–1^, respectively (Figure 3A).

In the DSC curves of the samples with compositions of 0.333 < *x*(PrBr_3_) < 1, in addition to the effect associated with the liquidus line, another endothermic effect can be observed at approximately 743 K (Figure 1C,D). The recorded transformation corresponds to another eutectic reaction in the studied system
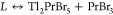
3

As in the previous case, the composition of the eutectic was determined from the intersection of the two straight lines of the Tammann diagram (Figure 3B) and was found to be *x*(PrBr_3_) = 0.551 ± 0.021, while the enthalpy associated with this eutectic reaction was equal to 19.3 ± 1.5 kJ·mol^–1^. Analysis of both Tammann diagrams confirms the existence of the compound Tl_2_PrBr_5_.

**Figure 4:**
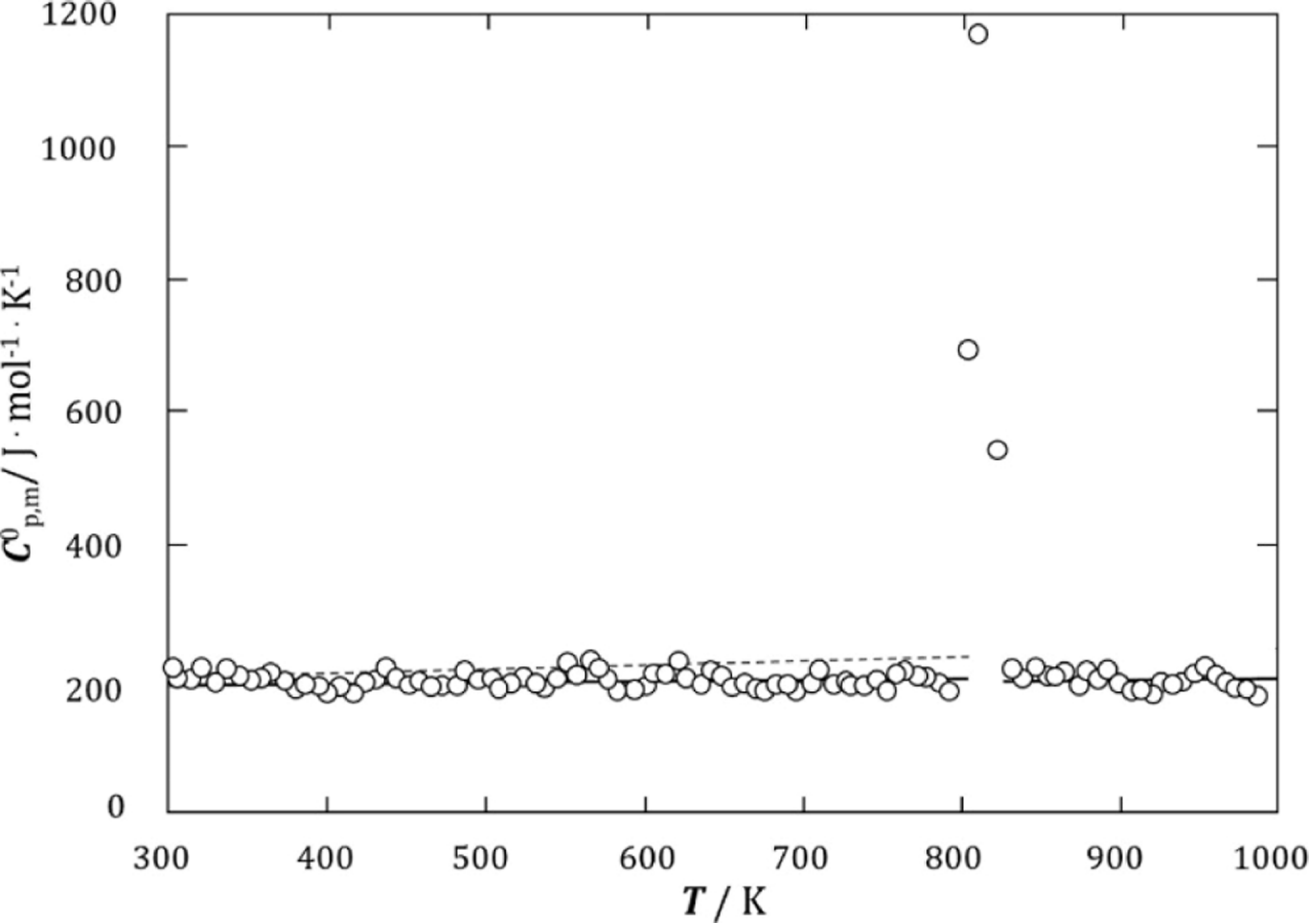
Molar heat capacity of Tl_2_PrBr_5_ (points—experimental results, solid lines—polynomial fitting of experimental results, and dotted line—theoretical molar heat capacity derived from the Neumann–Kopp rule).

The determined molar heat capacity as a function of temperature for the congruent melting compound Tl_2_PrBr_5_ is linear and increases slightly with increasing temperature (Figure 4)
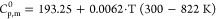
4

For the liquid phase, the molar heat capacity was approximated, assuming its independence from temperature and equal to 202.14 J·mol^–1.^ K^–1^. The experimental results obtained for the Tl_2_LnBr_5_ compound were further used to determine the thermodynamic dependencies (Table 3). The value of the molar heat of the compound, at 298.15 K, was determined by extrapolating the experimentally determined dependence. The entropy values of the compound at 298.15 K were determined by the Latimer method,^19^ using entropy contribution data modified by Spencer.^20^

**Table 3:** Thermodynamic Functions of Tl_2_PrBr_5_ at the Temperature Range from 298.15 to 1000 K

*T* (K)	*C*_*p*,m_^0^(*T*) (J·mol^–1^·K^–1^)	*S*_m_^0^(*T*) (J·mol^–1^·K^–1^)	–[*G*_m_^0^(*T*) – *H*^SER^ (298.15)]/*T* (J·mol^–1^·K^–1^)	*H*_m_^0^(*T*) – *H*^SER^ (298.15 K) (kJ·mol^–1^)
298.15	195.10	440.00	440.00	0.00
300	195.11	441.21	440.00	0.36
400	195.73	497.42	447.66	19.90
500	196.35	541.16	462.15	39.51
600	196.97	577.02	478.40	59.17
700	197.59	607.43	494.71	78.90
800	198.21	633.85	510.49	98.69
827	198.38	640.43	514.62	104.04
827	202.14	666.08	514.62	125.25
900	202.14	683.17	527.61	140.00
1000	202.14	704.47	544.25	160.23

## CALPHAD

The data obtained by the DSC method was used to perform phase diagram optimization using the CALPHAD method. Furthermore, CALPHAD data verification ensures the highest possible compatibility of thermodynamic properties of phases present in the studied system, so it is considered an equivalent technique to experiment.^1^ A detailed description of the optimization by the CALPHAD method and the software used has already appeared in previous work.^7^

Choosing a model to describe the liquid phase is crucial in optimizing the phase diagram. There are several different models, including mathematical models and those with physical significance, that account for the nature of the liquid phase. Spectroscopic studies,^21^ carried out on lanthanide halide–lithium halide systems, indicate that the liquid phase in this type of system is of an associative nature, with a dominant {3M^+^ + LnX_3_^3–^} associate. Given the similarity of lanthanide halide systems with thallium halide to systems with alkali metal halides, it was assumed that the liquid phase in the PrBr_3_–TlBr system would also be of associative liquid character with a dominant {3Tl^+^ + PrBr_3_^3–^} species. The stoichiometry of the associate is most often the same as the stoichiometry of the highest melting intermediate compound in the system, but spectroscopic studies^21^ indicate that when M_2_LnX_5_ is the only congruent compound in LnX_3_–MX systems (Ln = lanthanide, M = alkali metal, X = halide), {3M^+^ + LnX_3_^3–^} associate is nevertheless the most dominant individuum in the liquid phase. In our assessment, the model that best describes this type of liquid phase is the Associate Model with one associate.^22,23^ Details of the model and its key assumptions have been described in previous work.^7^

The liquid phase of the system considered in this work is described by the formula
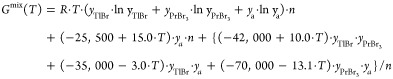
5where *y*_*i*_ is the molar fraction of all species in the liquid phase after the association process (associate, PrBr_3_, and TlBr), while *n* refers to the total number of moles in solution. The remaining coefficients (in J·mol^–1^) in this equation were determined by an iterative method with simultaneous control of the global minimum.

The temperature dependence of the Gibbs energy of formation for the Tl_2_PrBr_5_ compound was determined using the following formula
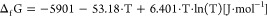
6

## Discussion

The determined PrBr_3_–TlBr phase diagram differs significantly from the results published by Molodkin et al.^16^ In the case of the diagram obtained in this work, the existence of only one stoichiometric compound Tl_2_PrBr_5_ was confirmed, while Molodkin et al.^16^ identified four compounds: Tl_2_PrBr_5_, TlPrBr_4_, TlPr_4_Br_13_, and TlPr_9_Br_28_. Additionally, the congruent melting temperature of the Tl_2_PrBr_5_ compound obtained in this study is 23 K higher than that obtained by other researchers.^16^ In the diagram determined by Molodkin et al.,^16^ one can also observe a very high concentration of phase transformations occurring in a relatively narrow temperature range.

The topology of the phase diagram^16^ is very peculiar as it does not resemble others known for systems of lanthanide(III) bromides with monovalent metal bromides.^2^ Literature data on such kinds of systems suggest the presence of phase tendencies resulting directly from the properties of lanthanide halides and monovalent metal halides.^18^ Consequently, the properties of LnX_3_–TlX systems should also change in a similar trend. Meanwhile, the topology of the diagrams of the binary systems EuCl_2_–TlCl, SmCl_3_–TlCl, LaBr_3_–TlBr, and CeBr_3_–TlBr^6,7,24^ does not indicate such a number of intermediate compounds and the multiplicity of phase transformations they undergo, as presented by the authors of the paper.^16^ The high complexity of the phase equilibria^16^ can result, for example, from incomplete homogenization of samples. In addition, DTA measurements were carried out by Molodkin et al.^16^ under heating/cooling conditions of 8–10 K·min^–1^, which may have been too high a rate concerning the mass of the samples (approximately 2 g). This would explain the additional effects of the kinetic transformations of metastable states, which are not observed in our phase diagram. The DTA thermal analysis method used in the 1970s by the authors of the previous paper^16^ is characterized by lower accuracy compared to the DSC used to obtain the results in this paper. The XRD analysis presented by Molodkin et al.^16^ also does not unambiguously confirm the existence of the compounds they identified. A unique set of diffraction lines does not represent the published diffractograms for the intermediate compounds. Moreover, in the literature,^16^ microscopic image results were discussed but not published. The authors published only the diffraction lines and not the full diffractographic spectrum from which one could deduce the possible presence of water in the samples, which is important from the point of view of the hygroscopicity of the tested compounds. Considering these factors, the phase equilibria in the PrBr_3_–TlBr binary system obtained in this work are more likely to be more accurate.

The dependence of the molar heat capacity on the temperature determined for the Tl_2_PrBr_5_ compound indicates its stability and the absence of phase transformation over the entire temperature range of its existence (298–822 K). The experimentally obtained dependence of *C*_*p*,m_(*T*) resembles the trend derived from the Neumann–Kopp rule, which is represented by the dashed line in Figure 4. The slight difference between the measured and theoretical values was included as a correction of the third coefficient in eq 6. This approximation is sufficient for this type of optimization.

**Figure 5:**
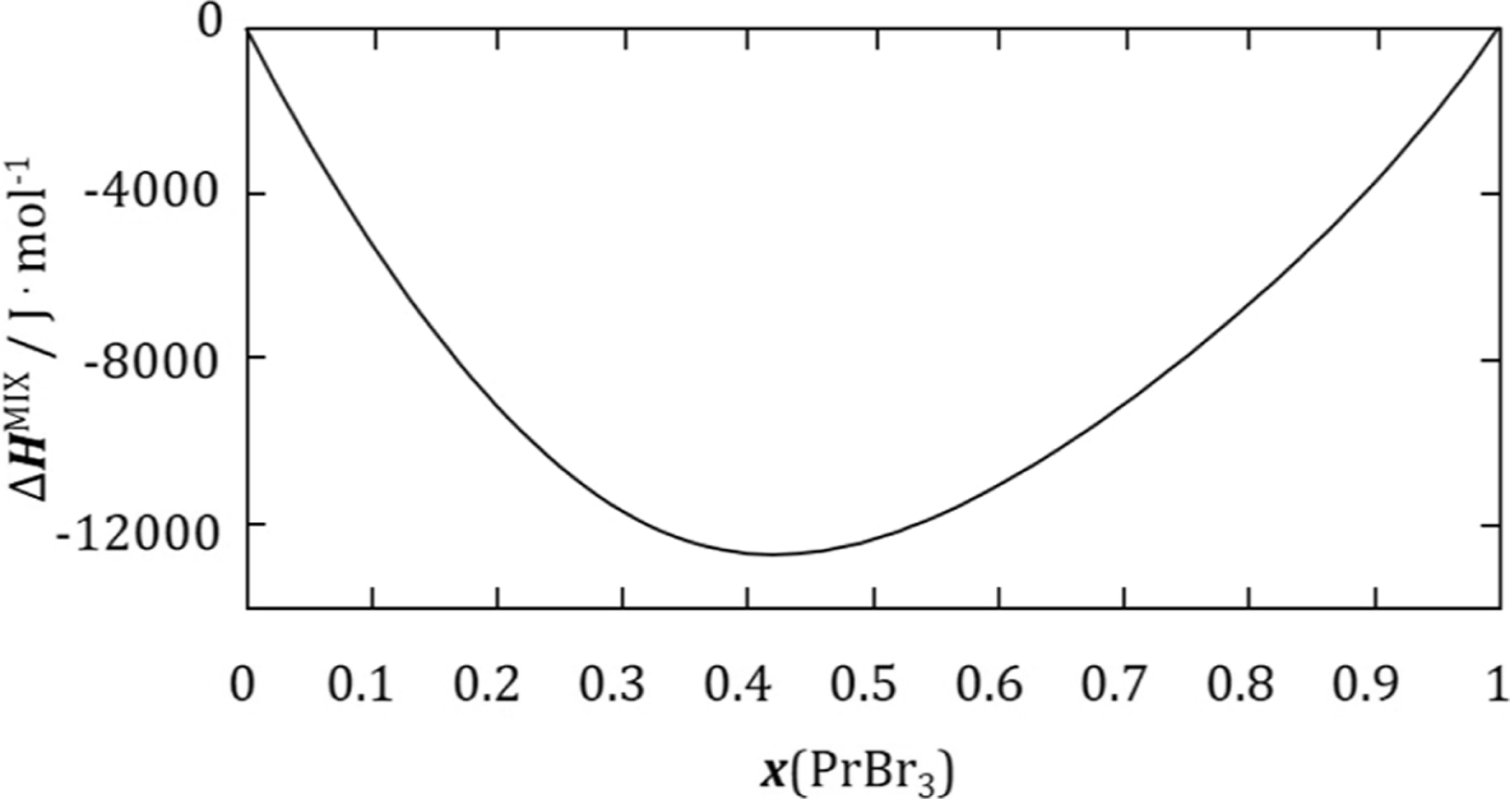
CALPHAD-optimized dependence of mixing enthalpy on composition at 1100 K.

**Figure 6:**
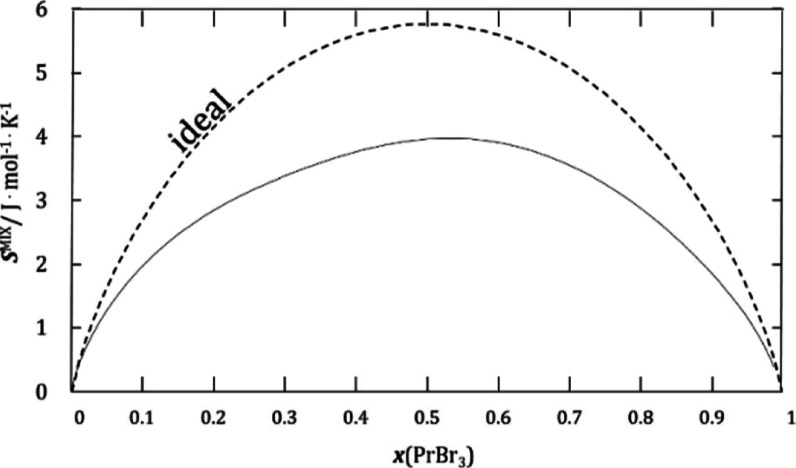
CALPHAD-optimized dependence of mixing entropy on composition at 1100 K; the dashed line represents the entropy curve for an ideal solution.

**Figure 7:**
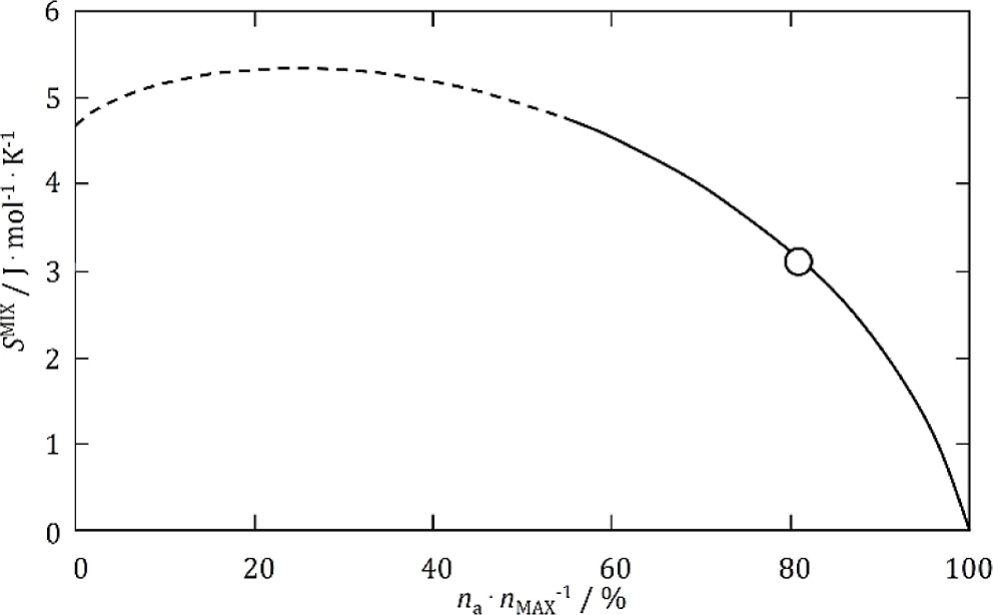
Configurational mixing entropy dependence on ratio describing an associate amount {3Tl^+^ + PrBr_6_^3–^} in its maximal amount at the mole fraction of PrBr_3_ equal to 0.250. The dashed line represents the region of ambiguous interpretation.

The obtained coupled phase diagram satisfactorily coincides with the data obtained by DSC measurements (Figure 2). In the results of the optimization carried out using the CALPHAD method, hitherto unmeasured composition dependencies of enthalpy and entropy of mixing were obtained (Figures 5 and 6). The asymmetric course of both of these dependencies is similar to analogous dependencies for lanthanide halide–lithium halide systems, suggesting an ordering in the liquid phase, confirming the existence of the associate. The abundance of the association is highest when the composition is consistent with the stoichiometry of the adopted associate.^22,23^ The entropy of mixing at this composition obtained using the CALPHAD procedure allows for calculating the amount of association in equilibrium with the pure components of the system through the mass action law of associate formation reaction
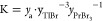
7
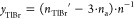
8
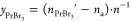
9
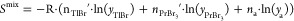
10where *K* is the equilibrium constant, *n*_TlBr_′ and *n*_PrBr_3__′ are a number of moles of pure system components after the association process. The values of all mole fractions were calculated iteratively. The value of the entropy of mixing in the composition *x*(PrBr_3_) = 0.250 (*S*^mix^ = 3.11 J·mol^–1^·K^–1^) for the system under consideration was correlated with the ratio of the amount of associate {3Tl^+^ + PrBr_6_^3–^} (*n*_a_) to its maximum possible abundance (*n*_max_) in this composition (Figure 7). In the case of this system, the content of the {3Tl^+^ + PrBr_3_^3–^} associate in the liquid phase was estimated to be at least 81%.

## Conclusions

The phase equilibria in the PrBr_3_–TlBr system were verified. The results obtained only allowed confirmation of the existence of one congruently melting Tl_2_PrBr_5_ compound, and from the research undertaken, it results that the phase diagram topology proposed by Molodkin et al.^16^ has an improbable course. The determined temperature dependence of the molar heat capacity and Gibbs energy of formation for compound Tl_2_PrBr_5_ indicates its thermal and thermodynamic stability over the entire range of existence. One result of the CALPHAD optimization method is the enthalpy and entropy dependence of mixing on composition. The computational techniques also allowed for a characterization of the liquid phase in the studied system and estimation of the {3Tl^+^ + PrBr_3_^3–^} associate content.

The results obtained in the present work can further serve as a basis for new applied studies and confirm the rationale for proceeding with the presented research here as the success of targeted studies is contingent on fundamental investigations.
